# Genomic and phenotypic description of the newly isolated human species *Collinsella bouchesdurhonensis* sp. nov.

**DOI:** 10.1002/mbo3.580

**Published:** 2018-06-13

**Authors:** Melhem Bilen, Mamadou Beye, Maxime Descartes Mbogning Fonkou, Saber Khelaifia, Frédéric Cadoret, Nicholas Armstrong, Thi Tien Nguyen, Jérémy Delerce, Ziad Daoud, Didier Raoult, Pierre‐Edouard Fournier

**Affiliations:** ^1^ Aix‐Marseille Université URMITE UM63, CNRS7278, IRD198 INSERM 1095 Assistance Publique‐Hôpitaux de Marseille Institut Hospitalo‐Universitaire Méditerranée‐Infection Faculté de médecine Marseille France; ^2^ Clinical Microbiology Department Faculty of Medicine and Medical sciences University of Balamand Amioun Lebanon; ^3^ Special Infectious Agents Unit King Fahd Medical Research Center King Abdulaziz University Jeddah Saudi Arabia

**Keywords:** *Collinsella bouchesdurhonensis*, culturomics, genome, pygmy, taxonomy

## Abstract

Using culturomics, a recently developed strategy based on diversified culture conditions for the isolation of previously uncultured bacteria, we isolated strain Marseille‐P3296^T^ from a fecal sample of a healthy pygmy female. A multiphasic approach, taxono‐genomics, was used to describe the major characteristics of this anaerobic and gram‐positive bacillus that is unable to sporulate and is not motile. The genome of this bacterium is 1,878,572 bp‐long with a 57.94 mol% G + C content. On the basis of these characteristics and after comparison with its closest phylogenetic neighbors, we are confident that strain Marseille‐P3296^T^ (=CCUG 70328 =  CSUR P3296) is the type strain of a novel species for which we propose the name *Collinsella bouchesdurhonensis* sp. nov.

## 1 INTRODUCTION

Over the past decade, metagenomics has been extensively adopted to enhance the description of the human gut microbiota (Gill et al., 2006; Ley, Turnbaugh, Klein, & Gordon, 2006; Ley et al., 2005). However, many detected DNA sequences cannot be assigned to as‐yet cultured microorganisms, suggesting that a significant part of the human gut microbiota content remains to be isolated and described (Rinke et al., 2013). Thus, the ability to culture and obtain pure bacterial colonies is mandatory for a better description, analysis, and correlation with health and diseases (Lagier et al., 2015). Stool samples are recognized as a representative model of the gut microbiota (Raoult & Henrissat, 2014). To improve bacterial isolation from these specimens, a strategy named culturomics was recently developed (Lagier, Armougom, Million et al., 2012; Lagier, Armougom, Mishra, et al., 2012; Lagier, El Karkouri, et al., 2012 Mishra, Lagier, Robert, Raoult, & Fournier, 2012; Seng et al., 2009). This method relies on culturing samples using diversified combinations of culture media, temperatures, atmospheres, and incubation times. All isolated colonies from a specimen are identified using matrix‐assisted laser desorption/ionization‐time of flight mass spectrometry (MALDI‐TOF MS). In case of identification's failure by MALDI‐TOF MS, 16S rRNA amplification, and sequencing is systematically performed for further phylogenetic analysis with closely related bacterial species (Lagier, Armougom, Mishra, et al. 2012; Lagier, El Karkouri,et al 2012; Mishra et al., 2012; Seng et al., 2009). Prior to the development of culturomics, only 688 bacteria were reported to be isolated from the human gut (Lagier et al., 2016). To date, culturomics has permitted the isolation of more than 1,000 distinct human gut bacterial species, including a significant number of novel species (Lagier et al., 2016). Using culturomics, we report the isolation of strain Marseille‐P3296^T^ from a human stool sample, which we believe to be the representative strain of a new *Collinsella* species and propose the name *Collinsella bouchesdurhonensis* (*C. bouchesdurhonensis*) sp. nov. The *Collinsella* genus was first described by Kageyama, Benno, & Nakase, (1999), after the reclassification of *Eubacterium aerofaciens* into a new genus on the basis of a high 16S rRNA gene sequence divergence with other members of the *Eubacterium* genus. In addition to the type species *C. aerofaciens* (Kageyama et al., 1999), the *Collinsella* genus currently contains *C. intestinalis* (Kageyama & Benno, 2000)*,C. massiliensis*(Padmanabhan et al., 2014)*, C. stercoris* (Kageyama & Benno, 2000), and *C. tanakaei* (Nagai, Watanabe, & Morotomi, 2010). Members of the *Collinsella* genus are gram‐positive anaerobic bacilli from the human gut microbiota that were suggested to play a role in health and diseases such as rheumatoid arthritis (Chen et al., 2016) and irritable bowel syndrome (Lee & Bak, 2011). They were also isolated from patients with Crohn's disease, ulcerative colitis, and colon cancer (Whitman et al., 2012).

Herein, we describe *C. bouchesdurhonensis* sp. nov. strain Marseille‐P3296^T^ (=CCUG 70328 =  CSUR P3296) using the taxono‐genomics approach.

## 2 MATERIAL AND METHODS

### 2.1 Ethics and sample collection

The feces donor is a healthy 50‐year‐old pygmy female from Congo. She gave an informed and signed consent. Samples were stored at the URMITE laboratory (Marseille, France) at −80°C for further analysis. The culturomics study was approved by the ethics committee of the Institut Fédératif de Recherche 48 under number 09–022.

### 2.2 Strain isolation

After being diluted with phosphate‐buffered saline (Life Technologies, Carlsbad, CA), stool samples were incubated in an anaerobic blood culture bottle (BD BACTEC^®^, Plus Anaerobic/F Media, Le Pont de Claix, France) supplemented with 5% sheep blood and 5% sterile‐filtered rumen at 37°C. Following initial growth in the blood culture vial, the bacterium was subcultured on 5% sheep blood–enriched Columbia agar (bioMérieux, Marcy l'Etoile, France).

### 2.3 Identification of colonies identification

Colony identification was performed by the mean of MALDI‐TOF MS as previously described (Elsawi et al., 2017; Lagier, El Karkouri, Nguyen, Armougom, Raoult, and Fournier, 2012). For strains that were not identified, 16S rRNA gene amplification and sequencing was performed as formerly done (Morel et al., 2015). Then, sequences were assembled and modified using the CodonCode Aligner software (http://www.codoncode.com) and a comparative analysis with the sequences of closely related species was performed using the BLAST software (http://blast.ncbi.nlm.nih.gov.gate1.inist.fr/Blast.cgi). A new species was considered when the similarity threshold between the 16S rRNA gene sequence of the understudied strain and its closest phylogenetic species with stranding in nomenclature was below 98.65% or considered as a new genus in case the similarity threshold was below 95% (Kim, Oh, Park, & Chun, 2014). The generated mass spectrum and 16S rRNA gene sequence were added to the UR‐MS and the EMBL‐EBI databases, respectively.

### 2.4 Growth conditions

To identify the optimal growth conditions, strain Marseille‐P3296^T^ was cultured under several temperature, atmosphere, pH, and salinity conditions. First, strain Marseille‐P3296^T^ was cultured and incubated under aerobic, anaerobic (GENbag anaer, bioMérieux) and microaerophilic (GENbag Microaer, bioMérieux) conditions on 5% sheep blood‐enriched Columbia agar (bioMérieux) at the following temperatures: 28, 37, 45, and 55°C. Furthermore, the halotolerance and acidity tolerance were estimated using 0, 5, 10, 50, 75, and 100 g/L NaCl concentrations and pH values of 6, 6.5, 7, and 8.5, respectively.

### 2.5 Morphological and biochemical assays

The biochemical characteristics of strain Marseille‐P3296^T^ were described using multiple API strips (ZYM, 20A and 50CH, bioMérieux), using bacteria that had been cultivated on 5% sheep blood‐enriched Columbia agar, in anaerobic atmosphere at 37°C for 24 hr. In addition, the sporulation ability was tested by exposing a bacterial suspension to a thermic shock (80°C) for 20 min. Gram staining and cell motility were observed under a 100X magnification using a DM1000 photonic microscope (Leica Microsystems, Nanterre, France). Finally, cell morphology was observed by means of a Tecnai G20 Cryo (FEI) transmission electron microscope as previously described (Elsawi et al., 2017).

### 2.6 FAME analysis

GC/MS was used for cellular fatty acid methyl ester (FAME) analysis after preparing two cell samples containing around 15 mg of bacterial biomass per tube and analysis was performed as previously described (Dione et al., 2016). Moreover, Elite 5‐MS column was used for FAME separation and checked by mass spectrometry (Clarus 500‐SQ 8 S, Perkin Elmer, Courtaboeuf, France). Then, the FAME mass spectral database (Wiley, Chichester, UK) and MS Search 2.0 operated with the Standard Reference Database 1A (NIST, Gaithersburg, USA) were used for FAME identification.

### 2.7 Antibiotic resistance profile

The antibiotic resistance profile of the strain was evaluated using the *E*‐test method and the following molecules: benzylpenecilin, amoxicillin, cefotaxime, ceftriaxone, imipenem, rifampicin, minocycline, tigecycline, amikacin, teicoplanin, vancomycin, colistin, daptomycin, and metronidazole (Biomerieux, France).

### 2.8 DNA extraction and genome sequencing

Strain Marseille‐P3296^T^ genomic DNA (gDNA) was extracted after subjecting it first to a mechanical treatment with the FastPrep BIO 101 instrument (Qbiogene, Strasbourg, France) using acid washed glass beads (Sigma) at a speed of 6.5 m/s for 90 s. Then, an incubation of 3 hr with lysozyme was done at 37°C for a DNA extraction assay using the EZ1 biorobot (Qiagen). The elution volume was set to 50 μl and the final gDNA concentration (25.8 ng/μl) was determined using a Qubit assay with the high sensitivity kit (Life technologies, Carlsbad, CA, USA).

The gDNA from strain Marseille‐P3296^T^ was sequenced using a MiSeq sequencer (Illumina Inc, San Diego, CA, USA) and the Mate Pair strategy as formerly described (Elsawi et al., 2017). The size of the DNA fragments obtained ranged from 1.5 kb to up to 11 kb with an optimal size at 7.933 kb. Additionally, a circularization of 600 ng of tagmented DNA was done with no size selection. Mechanical shearing of the circularized DNA was performed using the Covaris device S2 in T6 tubes (Covaris, Woburn, MA, USA) in order to obtain small DNA fragments with an optimal size of 981 bp.

High Sensitivity Bioanalyzer LabChip (Agilent Technologies) was used for libraries profile visualizations and a 19.28 nmol/L final concentration was measured. Normalization of the libraries was done at 2 nmol/L and pooled. Then, following denaturation, libraries were diluted to reach a concentration of 15 pmol/L. Libraries were loaded onto the reagent cartridge and then onto the instrument along with the flow cell. Cluster generation was done automatically and a single 2 x 251‐bp run was performed for sequencing. A total of 9.5 Gb information was acquired from a cluster density of 1,050 K/mm^2^ with a quality threshold of 92.5% (18,644,000 passing filter paired reads). Strain Marseille‐P3296^T^ index representation in this run was 7.78%. The 1,451,051 paired reads were trimmed and then assembled.

### 2.9 Genome assembly

The genome assembly was performed using an in‐house pipeline combining various softwares (Velvet (Zerbino & Birney, 2008), Spades (Bankevich et al., 2012), and Soap Denovo (Luo et al., 2012)), on trimmed (MiSeq and Trimmomatic softwares (Bolger, Lohse, & Usadel, 2014)) or untrimmed data (only MiSeq software). Gap reduction was performed using GapCloser tool for each assembly (Luo et al., 2012). Then, phage Phix was adapted for contamination detection and elimination. Finally, any scaffolds with a size lower than 800 bp or with a depth value less than 25% of the mean depth, were considered as potential contaminants and were eliminated. The best assembly was chosen based on multiple characteristics such as number of Ns, N50 and number of scaffolds. For the studied strain, the Velvet software gave the best assembly, with a mean depth coverage of 386.

### 2.10 Genome annotation

The Prodigal software (Hyatt et al., 2010) was used with default parameters for Open Reading Frames (ORFs) detection and any ORFs that are spanning a sequencing gap were excluded.

The Clusters of Orthologous Groups (COG) database was used for bacterial protein sequences detection using BLASTP as previously described (Elsawi et al., 2017). Also, transfer RNA genes were searched using the tRNAScanSE (Lowe & Eddy, 1997) software and RNAmmer tool was used for ribosomal RNA genes detection (Lagesen et al., 2007). Moreover, Phobius (Käll, Krogh, & Sonnhammer, 2004) was used for lipoprotein signal peptides and transmembrane helices detection. ORFans were identified based on the BLASTP results as previously determined (Elsawi et al., 2017). Finally, annotation was performed with the DAGOBAH software (Gouret et al., 2011) that contains the Figenix Librairies (Gouret et al., 2005).

### 2.11 16S rRNA phylogenetic analysis

Sequences of the strains to be considered in the phylogenetic analyses were obtained after performing a BLASTn search against the 16S rRNA database of “The All‐Species Living Tree” Project of Silva (Yilmaz et al., 2014). Sequences were aligned with CLUSTALW (Thompson, Higgins, Gibson, & Gibson, 1994) and MEGA software (Kumar, Tamura, & Nei, 1994) was used for phylogenetic inferences generation with the maximum‐likelihood method.

### 2.12 Genome comparison analysis

GenBank was used for retrieving the ORFeome, proteome, and complete sequences of the species being used for the comparative analyses. Nevertheless, the species were automatically recovered using Phylopattern (Gouret, Thompson, & Pontarotti, 2009) from the 16S rRNA tree. If no genome was available for a specific strain, a complete genome from another strain of the same species was used. If ORFeome and proteome were not predicted, Prodigal was used with default parameters to predict them from the genome sequence. ProteinOrtho was used for proteome analyses (Lechner et al., 2011). Then, AGIOS similarity scores were obtained with MAGI software for each couple of genomes (Ramasamy et al., 2014). These scores represent the mean value of nucleotide similarity between all couples of orthologous genes of the selected genomes. Proteome annotation was also performed for functional classes of predicted genes determination based on the clusters of orthologous groups of proteins (as was performed for genome annotation).

The comparison protocol was done with DAGOBAH (Gouret et al., 2011) which provided pipeline analysis, and using Phylopattern (Gouret et al., 2009) for tree manipulation.

## 3 RESULTS AND DISCUSSION

### 3.1 Strain identification

**Figure 1:**
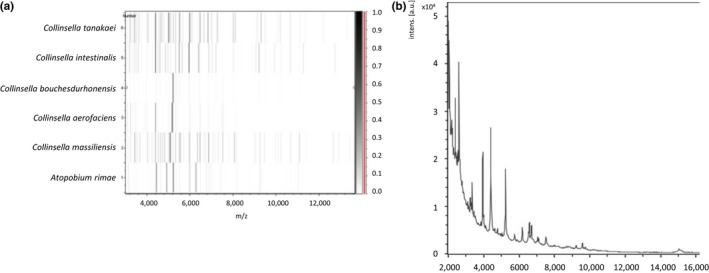
(a) Gel view comparing *Collinsella bouchedurhonensis* strain Marseille‐P3296^T^ to other species. This figure shows in a pseudo‐gel like format the raw spectra of the different strains shown on the left. The *x*‐axis shows the m/z value. The left *y*‐axis represents the running spectrum number obtained from subsequent spectra loading. The gray scale scheme code represents the intensity of the peaks and the *y*‐axis shows the correlation between the peak color and its intensity. (b) Reference mass spectrum representing *Collinsella bouchesdurhonensis* strain Marseille‐P3296^T^ acquired after analyzing 12 spectra

**Figure 2:**
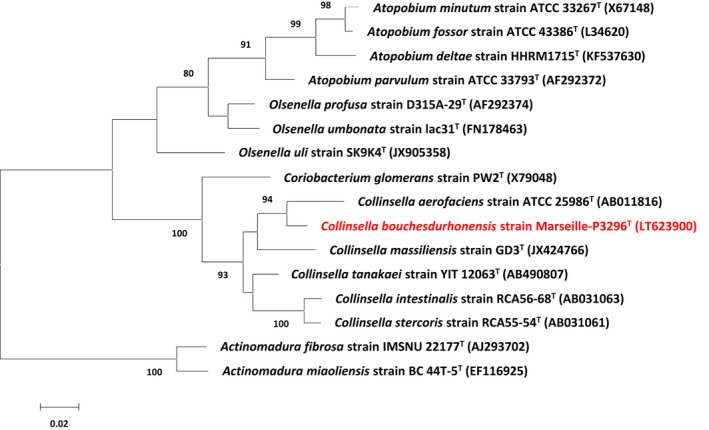
Phylogenetic tree representing the position of *Collinsella bouchesdurhonensis* strain Marseille‐P3296^T^ relative to other closely related species. The 16S rRNA Database of “The All‐Species Living Tree” Project of Silva (LTPs121) was used for sequence collection. The Muscle and FastTree softwares were used for sequence alignment and phylogenetic inferences with the maximum‐likelihood method, respectively. Numbers at the nodes are percentages of bootstrap values obtained by repeating the analysis 1,000 times to generate a majority consensus tree. Only values >70% are displayed. *Actinomadura fibrosa* strain IMSNU 22177^T^ and *Actinomadura miaoliensis* strain BC 44T‐5^T^ were used as outgroup

MALDI‐TOF MS could not identify strain Marseille‐P3296^T^. Hence, its type spectrum (Figure 1a) was added to the URMS database and compared to spectra from other closely related species (Figure 1b). The 16S rRNA gene sequence exhibited a 96.19% sequence similarity with *Collinsella aerofaciens* strain JCM10188^T^ (GenBank accession number AB011816), the phylogenetically closest species with a validly published name (Figure 2). Strain Marseille‐P3296^T^ exhibiting a 16S rRNA divergence greater than 1.35% with its closest phylogenetic neighbor (Kim et al., 2014), we investigated whether it possessed sufficient phenotypic and genomic differences with *C. aerofaciens* and other *Collinsella* species to be considered as a new species. The 16S rRNA sequence from strain Marseille‐P3296^T^ was submitted to GenBank with the number LT623900.

### 3.2 General biochemical and phenotypic characteristics of strain Marseille‐P3296^T^

Strain Marseille‐P3296^T^ is a strictly anaerobic bacterium, able to grow at temperatures between 37°C and 42°C but optimally at 37°C). This strain can sustain a pH range of 6–8.5 and NaCl concentrations up to 5 g/L. After 24 hr of culture on agar, colonies are white and smooth with a diameter of 0.1–0.5 mm. Cells are rods, gram‐negative with a mean width of 0.5 μm and a mean length of 2.6 μm (Table 1, Figures S1& S2), are not motile, are unable to sporulate and exhibit no catalase and oxidase activities.

**Table 1:** Classification and general characteristics of *C. bouchesdurhonensis* strain Marseille‐P3296^T^

Property	Term	References
Current classification	Domain: *Bacteria*	(Woese, Kandler, & Wheelis, 1990)
	Phylum: *Actinobacteria*	(Cavalier‐Smith, 2002)
	Class: *Actinobacteria*	(Stackebrandt, Rainey, & Ward‐Rainey, 1997)
	Order: *Coriobacteriales*	(Gupta, Chen, Adeolu, & Chai, 2013; Stackebrandt et al., 1997)
	Family: *Coriobacteriaceae*	(Gupta et al., 2013)
	Genus: *Collinsella*	(Kageyama et al., 1999)
	Species: *Collinsella bouchesdurhonensis*	(Bilen, Cadoret, Daoud, Fournier, & Raoult, 2016)
	Type Strain: Marseille‐P3296	
Gram stain	Negative	
Motility	Negative	
Cell shape	Bacillus	
Sporulation	Negative	
Optimum temperature	37°C	
Temperature range	37–42°C	

Strain Marseille‐P3296^T^ was susceptible to benzylpenecilin, amoxicillin, cefotaxime, ceftriaxone, imipenem, rifampicin, tigecycline, teicoplanin, vancomycin, and metronidazole (minimal inhibitory concentrations [μg/ml] of 0.006, 0.64, 0.125, 0.56, 0.12, 0.004, 0.19, 0.016, 1, and 0.064, respectively), but resistant to minocycline, amikacin, colistin, and daptomycin (4, >256, >256, and 2, respectively). Similar antibiotic susceptibility profiles were previously reported for *C. massiliensis* (Padmanabhan et al., 2014), *Collinsella aerofaciens, C. intestinalis,* and *C. tanakei* (Whitman et al., 2012)

Using API ZYM, strain Marseille‐P3296^T^ exhibited alkaline phosphatase, valine arylamidase, esterase lipase (C8), α‐fucosidase, esterase (C4), αchymotrypsin, leucine arylamidase, and acid phosphatase activities but was negative for β‐glucosidase, lipase (C14), trypsin, naphtol‐AS‐BI‐phosphohydrolase, N‐acetyl‐β‐glucosaminidase, cystine arylamidase, α‐ mannosidase, β‐ galactosidase, α‐galactosidase, β‐glucuronidase, andα‐glucosidase.

With the mean of API 50 CH, fermentation was observed in the presence of methyl‐αD‐glucopyranoside, d‐galactose, potassium gluconate, D‐mannose, D‐arabitol, D‐fructose, L‐sorbose, gentiobiose, D‐melezitose, D‐glucose, D‐lactose, D‐trehalose, glycerol, D‐raffinose, D‐turanose, D‐maltose, D‐tagatose, D‐xylose, and D‐saccharose (sucrose). In contrast, no fermentation was obtained with erythritol, L‐arabinose, D‐ribose, D‐adonitol, L‐xylose, dulcitol, D‐arabinose, inositol, L‐rhamnose, methyl‐βD‐xylopyranoside, D‐melibiose, inulin, methyl‐D‐mannopyranoside, glycogen, D‐fucose, starch, xylitol, D‐lyxose, potassium 5‐ketogluconate, potassium 2‐ketogluconate, L‐fucose, and L‐arabitol.

Moreover, when testing strain Marseille‐P3296^T^ with API 20A, it could not form indole and was urease‐negative. Additionally, acidification of glucose, lactose, maltose, salicin, arabinose, xylose, cellobiose, mannitol, mannose, rhamnose, melezitose, saccharose, sorbitol, and trehalose was observed, in contrast to glycerol and raffinose. Cells were also gelatin‐positive, but β‐glucosidase‐negative. A comparison of phenotypic and biochemical features between compared species is presented in Table 2.By comparison with other studied species, Strain Marseille‐P3296^T^ differed in a combination of production of N‐acetyl‐β‐glucosamine, absence of β‐galactosidase activity, and acidification of D‐mannitol. In contrast, all compared *Collinsella* species included Gram‐positive bacilli that were unable to sporulate and did neither exhibit any urease activity nor could produce acid from L‐arabinose. The G + C content of *C. bouchesdurhonensis, C. massiliensis, C. aerofaciens, C. intestinalis, and C, stercoris* is 57.94, 65.8, 61, 61.2, and 64.4 mol %, respectively (Table 2).

**Table 2:** Differential phenotypic characteristics of *C. bouchesdurhonensis* strain Marseille‐P3296^T^, *C. aerofaciens*,* C. massiliensis*,* C. intestinalis,* and *C. stercoris*

Properties	*Collinsella bouchesdurhonensis*	*Collinsella Massiliensis*	*Collinsella aerofaciens*	*Collinsella intestinalis*	*Collinsella stercoris*
Cell length (μm)	2,6	1,19	1.2–4.3	1.3–2.4	1.2–2.2
Oxygen requirement	Strictly anaerobic	Strictly anaerobic	Strictly anaerobic	Strictly anaerobic	Strictly anaerobic
Gram stain	Positive	Positive	Positive	Positive	Positive
Motility	−	−	Na	−	−
Endospore formation	−	−	−	−	−
Indole	−	−	Na	Na	Na
Production of					
Alkaline phosphatase	+	+	−	+	+
Catalase	−	−	Na	Na	Na
Oxidase	−	−	Na	Na	Na
Urease	−	−	−	−	−
β‐galactosidase	−	+	+	Na	Na
N‐acetyl‐β‐glucosamine	+	−	Na	Na	Na
Acid from					
L‐Arabinose	−	−	−	−	−
D‐Ribose	−	−	−	−	+
D‐Mannose	+	−	+	+	+
D‐Mannitol	+	−	−	−	−
D‐glucose	+	−	+	+	+
D‐fructose	+	−	+	+	+
D‐maltose	+	−	+	+	−
D‐lactose	+	−	+	+	−
G + C content (mol%)	57,94	65,8	61	61,2	64,4
Habitat	Human gut	Human gut	Human gut	Human gut	Human gut

The major fatty acid of strain Marseille‐P3296^T^ was the unsaturated 9‐octadecanoic acid (C18:1n9, 35%). Several abundant saturated fatty acids were also identified, including hexadecanoic acid (C16:0, 30%), octadecanoic acid (C18:0, 7%), tetradecanoic acid (C14:0, 13%), and dodecanoic acid (C12:0, 6%) (Table S1). By comparison, *C. aerofaciens, C. intestinalis, and C. stercoris* possessed octadecanoic acid as major fatty acid (Nagai et al., 2010).

### 3.3 Genome characteristics of strain marseille‐P3296^T^

The genome of strain Marseille P3296^T^ is 1,878,572‐bp long with a 57.94 mol% G + C content. It is made of 15 scaffolds (for a total of 24 contigs). Of the 1,711 predicted genes, 51 are RNAs (1 16S rRNA, 1 5S rRNA, 48 tRNA genes, and 1 23S rRNA) and 1,660 are protein‐coding genes. Fifty‐two genes are detected as ORFans (3,17%) and 1,348 genes (82,15%) are assigned a putative function (by BLAST against nr or COGs). The 193 genes (11.76%) remaining are defined as hypothetical proteins (Table S2). A graphical representation of the genome from strain Marseille‐P3296^T^ is presented in Figure S3. Table S4 details the distribution of genes into COG functional categories.

### 3.4 Comparative analysis between the genomes of strain Marseille‐P3296^T^ and closely related species

The draft genome sequence of strain Marseille‐P3296^T^ was compared to those of *Collinsella aerofaciens* (*C. aerofaciens*) (AAVN00000000), *C. tanakaei* (ADLS00000000), *C. stercoris* (ABXJ00000000), *C. intestinalis* (ABXH00000000), *C. massiliensis* (NZ_CAPI01000000), *Olsenella uli* (*O. uli*, NR_075775.1), *Atopobium parvulum* (*A. parvulum*, NC_013203.1), *A. rimae* (ACFE00000000), *A. minutum* (AGXC00000000), and *Coriobacterium glomerans* (*C. glomerans*, NC_015389.1*)*.

The draft genome of strain Marseille‐P3296^T^ (1,88 Mb) is smaller than those of *C. aerofaciens*,* C. tanakaei*,* C. stercoris*,* O. uli*,* C. massiliensis,* and *C, glomerans* (2,44, 2,48, 2,40, 2,05, 2,33, and 2,12 Mb, respectively), but larger than those of *C. intestinalis, A. minutum, A. rimae,* and *A. parvulum* (1,8, 1,71, 1,63, and 1,54 MB, respectively). Also, the G+C content of strain Marseille‐P3296^T^ (57.9), is smaller than those of *C. massiliensis*,* C. aerofaciens*,* C. intestinalis, C. tanakaei, C. stercoris*,* O. uli,* and *C. glomerans* (65.8, 60.5, 62.5, 60.2, 63.2, 64.7, and 60.4%, respectively), but larger than those of *A. minutum, A. rimae,* and *A. parvulum* (48.9, 49.3, and 45.7%, respectively) (Table S3).

As for the gene content of strain Marseille‐P3296^T^ (1,711), it is smaller than those of *C. massiliensis*,* C. aerofaciens, C. intestinalis, C. tanakaei, C. stercoris*,* O. uli, and C. glomerans* (2,046, 2,222, 1,626, 2,258, 2,106, 1,827, and 1,859, respectively), but larger than those of *A. minutum, A. rimae,* and *A. parvulum* (1,593, 1,523, and 1,411, respectively). The distribution of functional classes of predicted genes of strain Marseille‐P3296^T^ according to the COGs database is presented in Figure S4. The distribution was similar in all studied genomes.

Strain Marseille‐P3296^T^ shared the highest number of orthologous proteins with *C. tanakaei* (988). It also shared 971, 964, 890, and 860 orthologous proteins with *C. intestinalis*,* C. aerofaciens*,* C. massiliensis,* and *C. stercoris,* respectively, and lower numbers of orthologous proteins with species that belonged to other compared genera (Table S5). Moreover, in an effort to determine the degree of genetic relatedness of strain Marseille‐P3296^T^ with, and among *Collinsella* species, we used two parameters, AGIOS and dDDH. Within the *Collinsella* genus, excluding strain Marseille‐P3296^T^, AGIOS values ranged from 72.77% between *C. aerofaciens* and *C. tanakaei* to 81.12% between *C. stercoris* and *C. intestinalis* (Table S5). When compared to other *Collinsella* species, strain Marseille‐P3296^T^ exhibited AGIOS values ranging from 71.57% with *C. massiliensis* to 74.07% with *C. aerofaciens* (Table S5). In addition, within the *Collinsella* genus, excluding strain Marseille‐P3296^T^, dDDH values ranged from 22.1% between *C. aerofaciens* and *C. massiliensis* to 24.8% between *C. stercoris* and *C. tanakaei* (Table S6). When compared with other *Collinsella* species, strain Marseille‐P3296^T^ exhibited dDDH values ranging from 21.9% with *C. massiliensis* to 25% with *C. aerofaciens* (Table S6). For both parameters, strain Marseille‐P3296^T^ exhibited genomic similarity values in the range of those observed between other *Colinsella* species. In addition, the dDDH values lower than the 70% threshold that delimits bacterial species are consistent with the 16S rRNA sequence comparison and support the classification of strain Marseille‐P3296^T^ within a new species (Tindall, Rosselló‐Móra, Busse, Ludwig, & Kämpfer, 2010; Wayne, 1988).

## 4 CONCLUSION

On the basis of genomic, phenotypic, and biochemical features, we suggest the creation of a new species, *Collinsella bouchesdurhonensis* sp. nov. Strain Marseille‐P3296^T^ is the type strain of *C. bouchesdurhonensis* sp. nov.

### 4.1 Description of *Collinsella bouchesdurhonensis* sp. nov

*Collinsella bouchesdurhonensis* (bou.che.du.rho.nen'sis. N.L. fem. adj. *bouchesdurhonensis*, referring to Bouches‐du‐Rhône, the name of the department around Marseille, France, where this study was performed and strain Marseille‐P3296^T^ was isolated).

Strain Marseille‐P3296^T^ is a strictly anaerobic bacterium, able to grow at temperatures between 37°C and 42°C but optimally at 37°C). This strain can sustain a pH range of 6–8.5 and up to 5% NaCl concentration.

The species exhibits alkaline phosphatase, valine arylamidase, esterase lipase (C8), α‐fucosidase, esterase (C4), αchymotrypsin, leucine arylamidase, and acid phosphatase activities. Fermentation is observed in the presence of methyl‐ αD‐glucopyranoside, D‐galactose, potassium gluconate, D‐mannose, D‐arabitol, D‐fructose, L‐sorbose, gentiobiose, D‐melezitose, D‐glucose, D‐lactose, D‐trehalose, glycerol, D‐raffinose, D‐turanose, D‐maltose, D‐tagatose, D‐xylose, and D‐saccharose (sucrose). Strain Marseille‐P3296^T^ cannot form indole and is urease‐negative. Additionally, acidification of glucose, lactose, maltose, salicin, arabinose, xylose, cellobiose, mannitol, mannose, rhamnose, melezitose, saccharose, sorbitol, and trehalose is observed. The major fatty acid is 9‐octadecenoic acid.

The genome size 1,878,572‐bp long with a 57.94 mol% G + C content. The 16S rRNA and genome sequences of *C. bouchesdurhonensis* sp. nov. are deposited in EMBL‐EBI under accession numbers LT623900 and FTLD00000000, respectively. The type strain is Marseille‐P3296^T^ (=CSURP3296 = CCUG70328) and was isolated from the stool sample of a healthy 50‐year‐old pygmy woman from Congo.

## CONFLICTS OF INTEREST

No conflicts of interest are to be declared.
