# EXPRESSION OF CONCERN: Genomic and Phenotypic Description of the Newly Isolated Human Species *Collinsella bouchesdurhonensis* sp. nov.

**DOI:** 10.1002/mbo3.70246

**Published:** 2026-02-19

## Abstract

**EXPRESSION OF CONCERN:** M. Bilen, M. Beye, M.D.M. Fonkou, S. Khelaifia, F. Cadoret, N. Armstrong, T.T. Nguyen, J. Delerce, Z. Daoud, D. Raoult, and P.‐E. Fournier, “Genomic and Phenotypic Description of the Newly Isolated Human Species *Collinsella bouchesdurhonensis* sp. nov.,” *Microbiology Open* 7, no. 5 (2018): e00580, https://doi.org/10.1002/mbo3.580.

This Expression of Concern is for the above article, published online on 13 June 2018 in Wiley Online Library (wileyonlinelibrary.com), and has been issued by John Wiley & Sons Ltd. The Expression of Concern has been agreed due to questions raised about the study's adherence to French legal and ethical requirements for research involving human subjects, including questions regarding proper informed consent for research involving vulnerable populations. A question has also been raised by a third party about whether this particular article by the authors reports a “newly isolated” microbial strain that is in fact a “novel species.” The investigation into all of these concerns is ongoing. Therefore, the journal has decided to issue an Expression of Concern to inform and alert readers.

