# Supplementary material for: Genomic and phenotypic description of the newly isolated human species Collinsella bouchesdurhonensis sp. nov
Source: Microbiologyopen. 2018 Jun 13;7(5):e00580. doi: 10.1002/mbo3.580 (PMC6182551; doi:10.1002/mbo3.580)

**Supplementary Figure 1.** Gram staining of *Collinsella bouchesdurhonensis* strain Marseille-P3296^T^.


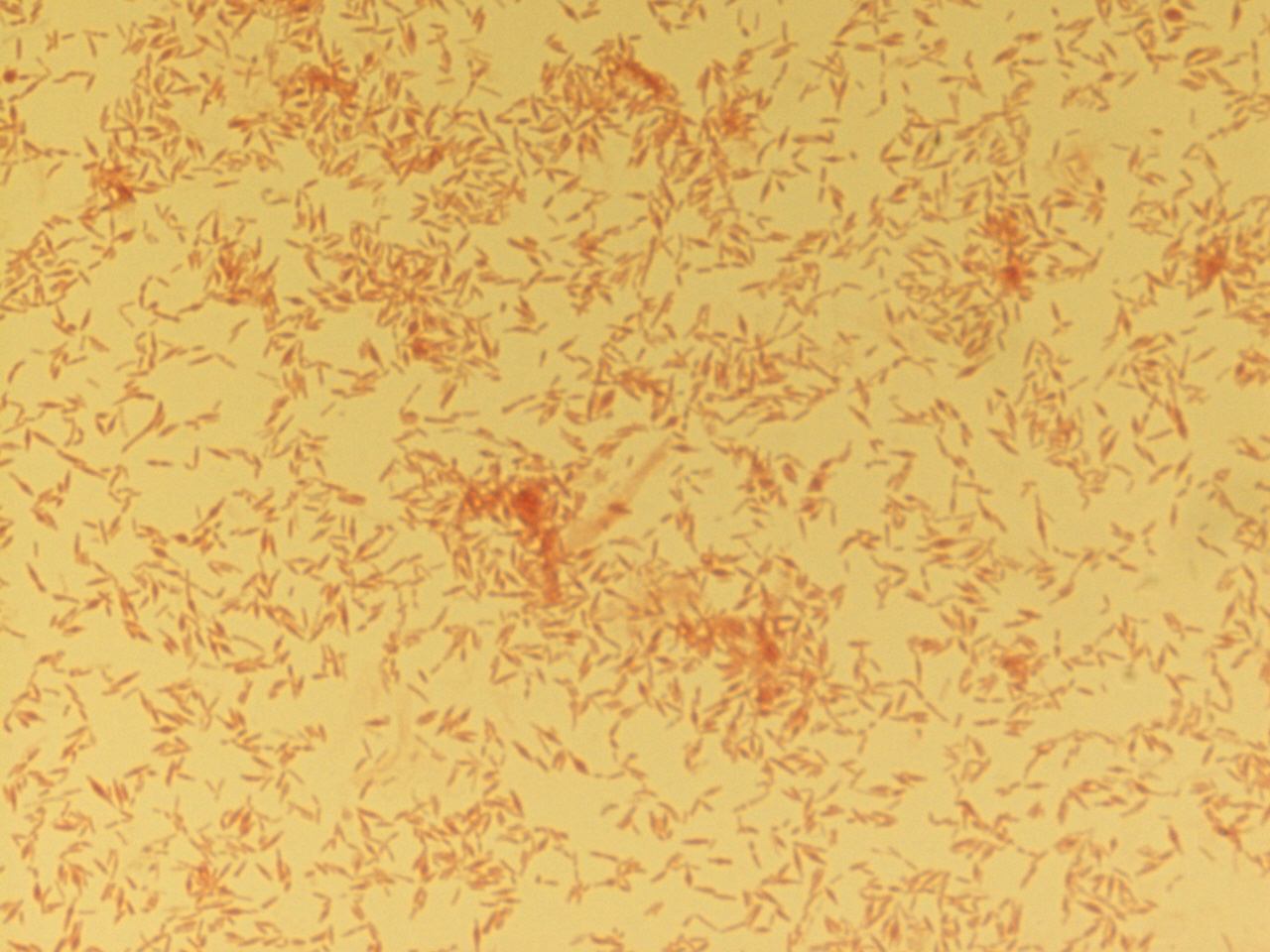


**Supplementary Figure 2.** Electron micrographs of *Collinsella bouchesdurhonensis* strain Marseille-P3296^T^ using a Tecnai G20, at an operating voltage of 200keV. Scale bar = 500 nm.


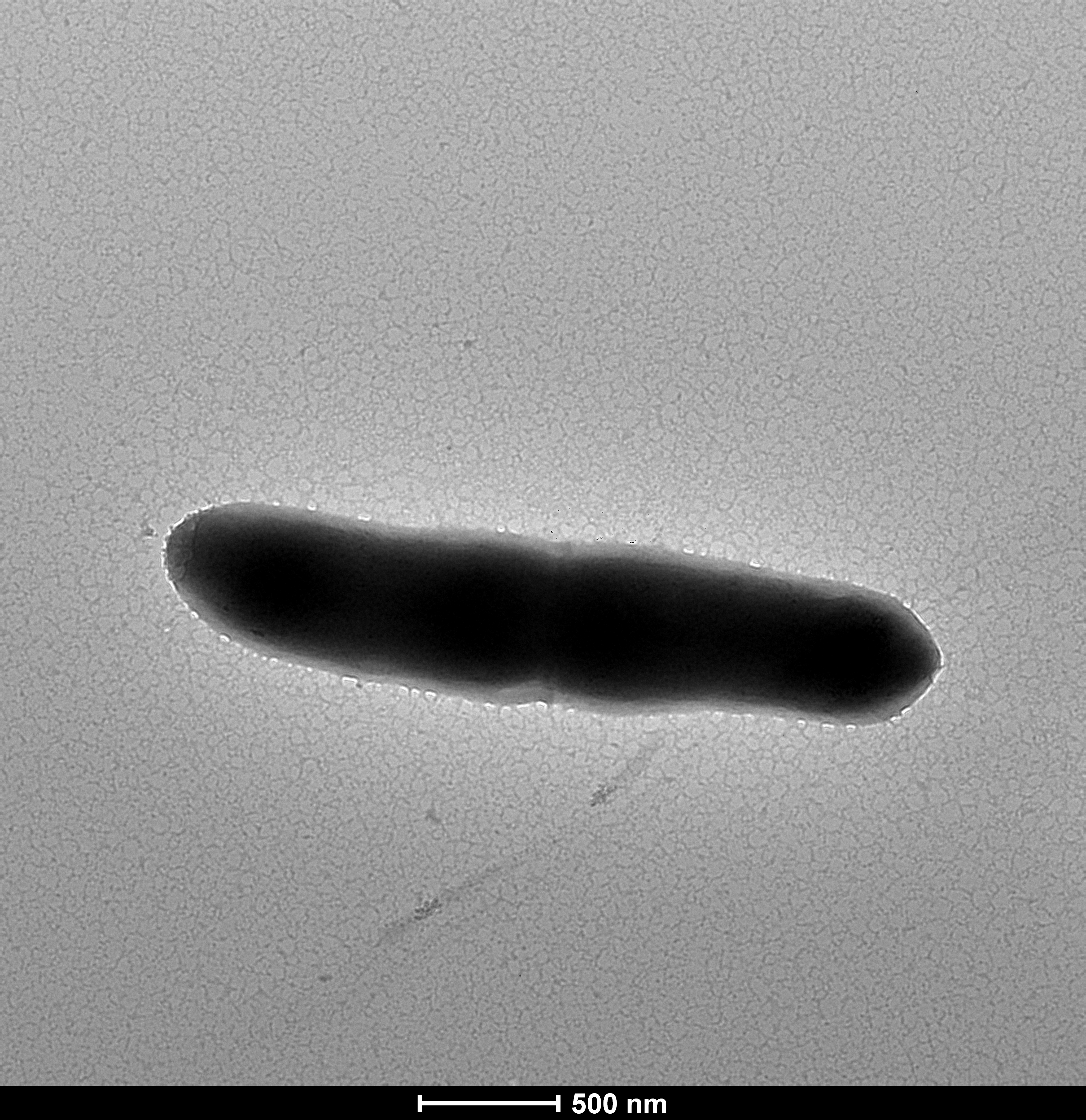


**Supplementary Figure 3.** Circular representation of the *Collinsella bouchesdurhonensis* strain Marseille-P3296^T^ genome. From the outside in: Contigs (red / grey), COG category of genes on the forward strand (three circles), genes on forward strand (blue circle), genes on the reverse strand (red circle), COG category on the reverse strand (three circles), G+C content.


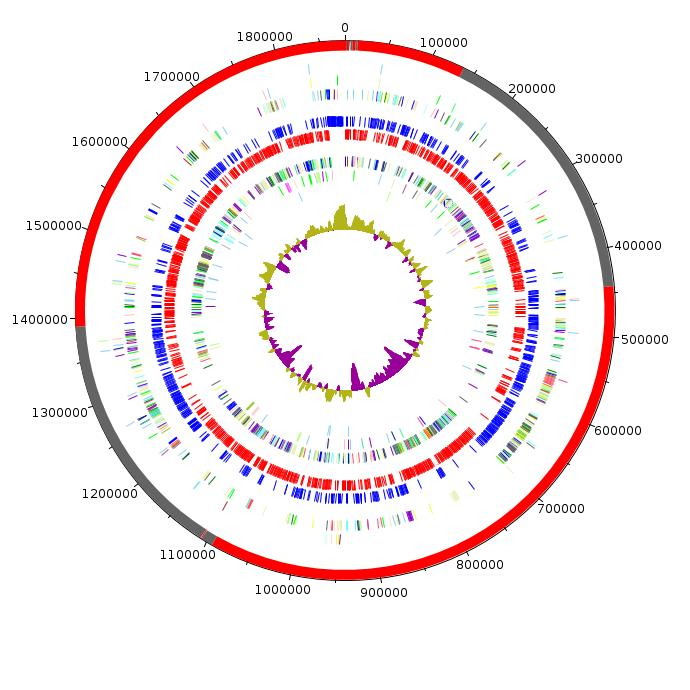


**Supplementary Figure 4.** Functional classes of predicted genes for *Collinsella bouchesdurhonensis* strain Marseille-P3296^T^ based on the clusters of orthologous groups of proteins.


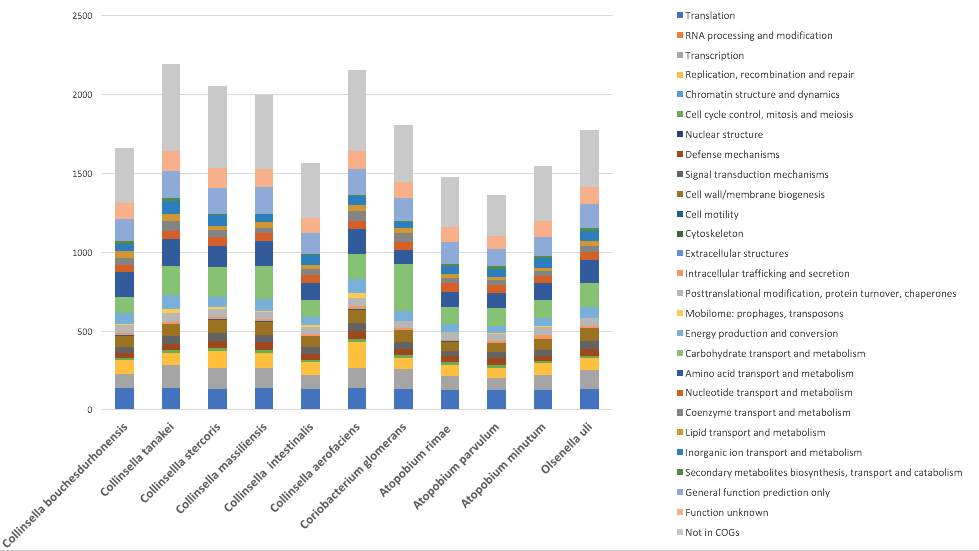

Supplement: Supplementary file 1 [file MBO3-7-e00580-s001.docx]
