# Supplementary material for: Genomic and phenotypic description of the newly isolated human species Collinsella bouchesdurhonensis sp. nov
Source: Microbiologyopen. 2018 Jun 13;7(5):e00580. doi: 10.1002/mbo3.580 (PMC6182551; doi:10.1002/mbo3.580)
Supplement: Supplementary file 2 [file MBO3-7-e00580-s002.docx]

**Supplementary Table 1.** Cellular fatty acid composition of *C. bouchesdurhonensis* strain Marseille-P3296^T^.

| Fatty acids | Name | Mean relative % ^a^ |
| --- | --- | --- |
| 18:1n9 | 9-Octadecenoic acid | 34.7 ± 2.4 |
| 16:00 | Hexadecanoic acid | 29.5 ± 1.8 |
| 14:00 | Tetradecanoic acid | 13.3 ± 0.8 |
| 18:00 | Octadecanoic acid | 6.8 ± 0.2 |
| 12:00 | Dodecanoic acid | 6.0 ± 1.2 |
| 18:1n3 | 15-Octadecenoic acid | 3.8 ± 0.3 |
| 18:2n6 | 9,12-Octadecadienoic acid | 2.7 ± 0.1 |
| 15:00 | Pentadecanoic acid | 1.3 ± 0.1 |
| 15:0 anteiso | 12-methyl-tetradecanoic acid | TR |
| 15:0 iso | 13-methyl-tetradecanoic acid | TR |
| 10:00 | Decanoic acid | TR |

^a^ Mean peak area percentage ; TR = trace amounts < 1 %

**Supplementary Table 2.** Genomic characteristics of *C. bouchesdurhonensis* strain Marseille-P3296^T^.

|  | Number | Percent^a^ |
| --- | --- | --- |
| Size (bp) | 1,878,572 | 100 |
| Number of G+C | 1,086,539 | 57.94 |
| Number total of genes | 1,711 | 100 |
| Number total of protein genes | 1,660 | 97.04 |
| Number total of RNA Genes | 51 | 2.96 |
| Number total of tRNA Genes | 48 | 2.84 |
| Number total of rRNA (5S, 16S, 23S) Genes | 3 | 0.12 |
| Coding sequence size | 1,635,450 | 87.06 |
| Coding sequence gene protein size | 1,630,065 | 86.77 |
| Coding sequence tRNA gene size | 3,773 | 0.2 |
| Coding sequence (5S, 16S, 23S) gene size | 1,612 | 0.09 |
| Number of protein coding gene | 1,641 | 100 |
| Number of protein associated to COGs | 1,132 | 68.98 |
| Number of protein associated to orfan | 52 | 3.17 |
| Number of protein with peptide signal | 106 | 6.46 |
| Number of gene associated to resistance genes | 1 | 0.06 |
| Number of gene associated to PKS or NRPS | 4 | 0.24 |
| Number of genes associated to virulence | 302 | 18.4 |
| Number of protein with TMH | 342 | 20.84 |

^a^The total is based on either the size of the genome in base pairs or the total number of protein- coding genes in the annotated genome

**Supplementary Table 3**. Genome comparison between *C. bouchesdurhonensis* strain Marseille-P3296^T^ and closely related species

**Supplementary Table 4.** Number of genes associated with the 25 general COG functional categories.

| **Code** | **Value** | **% of total** | **Description** |
| --- | --- | --- | --- |
| **[J]** | 139 | 8.37 | Translation |
| **[A]** | 0 | 0 | RNA processing and modification |
| **[K]** | 91 | 5.48 | Transcription |
| **[L]** | 86 | 5.2 | Replication, recombination and repair |
| **[B]** | 0 | 0 | Chromatin structure and dynamics |
| **[D]** | 16 | 0.96 | Cell cycle control. Mitosis and meiosis |
| **[Y]** | 0 | 0 | Nuclear structure |
| **[V]** | 30 | 1.81 | Defense mechanisms |
| **[T]** | 39 | 2.35 | Signal transduction mechanisms |
| **[M]** | 71 | 4.28 | Cell wall/membrane biogenesis |
| **[N]** | 3 | 0.18 | Cell motility |
| **[Z]** | 0 | 0 | Cytoskeleton |
| **[W]** | 0 | 0 | Extracellular structures |
| **[U]** | 16 | 0.96 | Intracellular trafficking and secretion |
| **[O]** | 48 | 2.89 | Posttranslational modification, protein turnover, chaperones |
| **[X]** | 3 | 0.18 | Mobilome: prophages, transposons |
| **[C]** | 73 | 4.4 | Energy production and conversion |
| **[G]** | 104 | 6.27 | Carbohydrate transport and metabolism |
| **[E]** | 158 | 9.52 | Amino acid transport and metabolism |
| **[F]** | 45 | 2.71 | Nucleotide transport and metabolism |
| **[H]** | 44 | 2.65 | Coenzyme transport and metabolism |
| **[I]** | 39 | 2.35 | Lipid transport and metabolism |
| **[P]** | 50 | 3.01 | Inorganic ion transport and metabolism |
| **[Q]** | 14 | 0.84 | Secondary metabolites biosynthesis, transport and catabolism |
| **[R]** | 144 | 8.67 | General function prediction only |
| **[S]** | 97 | 5.84 | Function unknown |
| **-** | 350 | 21.08 | Not in COGs |

**Supplementary Table 5.** AGIOS values (lower left) and numbers of orthologous genes (upper right) obtained by pairwise genomic comparisons. Bold numbers indicate the numbers of gene per genome. AP: *Atopobium parvulum*, CM: *Collinsella massiliensis*, CA: *Collinsella aerofaciens*, CB: *Collinsella bouchesdurhonensis*, CG: *Coriobacterium glomerans*, AR: *Atopobium rimae*, CI: *Collinsella intestinalis*, OU: *Olsenella uli,* CT: *Collinsella tanakaei*, CS: *Collinsella stercoris*, AM: *Atopobium minutum.*

**Supplementary Table 6.** Pairwise genomic comparison of *C. bouchesdurhonensis* strain Marseille-P3296^T^ with other species using the GGDC software, formula 2 (dDDH estimates based on identities over HSP length), upper right. The confidence intervals indicate the inherent uncertainty in estimating dDDH values from intergenomic distances based on models derived from empirical test data sets. AP: *Atopobium parvulum*, CM: *Collinsella massiliensis*, CA: *Collinsella aerofaciens*, CB: *Collinsella bouchedurhonensis*, CG: *Coriobacterium glomerans*, AR: *Atopobium rimae*, CI: *Collinsella intestinalis*, OU: *Olsenella uli,* CT: *Collinsella tanakaei* , CS: *Collinsella stercoris* , AM: *Atopobium minutum*.
